# Supplementary material for: Modeling the joint distribution of firm size and firm age based on grouped data
Source: PLoS One. 2020 Jul 15;15(7):e0235282. doi: 10.1371/journal.pone.0235282 (PMC7363102; doi:10.1371/journal.pone.0235282)
Supplement: Attachment — Submitted filename: P2P_Response.docx [file pone.0235282.s001.docx]

*We would like to thank the editor and the two reviewers for their thoughtful comments and efforts towards improving our manuscript. In the following, we provide and highlight the point-to-point responses in* **bold and blue** *font.*

| **Date:** | May 21 2020 02:30AM |
| --- | --- |
| **To:** | "Chen Ge" 174391437@qq.com |
| **From:** | "PLOS ONE" plosone@plos.org |
| **Subject:** | PLOS ONE Decision: Revision required [PONE-D-20-08858] |

PONE-D-20-08858
Modeling the Joint Distribution of Firm Size and Firm Age Based on Grouped Data
PLOS ONE

Dear Dr. Ge,

Thank you for submitting your manuscript to PLOS ONE. After careful consideration, we feel that it has merit but does not fully meet PLOS ONE’s publication criteria as it currently stands. Therefore, we invite you to submit a revised version of the manuscript that addresses the points raised during the review process.

We would appreciate receiving your revised manuscript by Jul 05 2020 11:59PM. When you are ready to submit your revision, log on to https://www.editorialmanager.com/pone/ and select the 'Submissions Needing Revision' folder to locate your manuscript file.

If you would like to make changes to your financial disclosure, please include your updated statement in your cover letter.

To enhance the reproducibility of your results, we recommend that if applicable you deposit your laboratory protocols in protocols.io, where a protocol can be assigned its own identifier (DOI) such that it can be cited independently in the future. For instructions see: <http://journals.plos.org/plosone/s/submission-guidelines#loc-laboratory-protocols>

Please include the following items when submitting your revised manuscript:

- A rebuttal letter that responds to each point raised by the academic editor and reviewer(s). This letter should be uploaded as separate file and labeled 'Response to Reviewers'.
- A marked-up copy of your manuscript that highlights changes made to the original version. This file should be uploaded as separate file and labeled 'Revised Manuscript with Track Changes'.
- An unmarked version of your revised paper without tracked changes. This file should be uploaded as separate file and labeled 'Manuscript'.

Please note while forming your response, if your article is accepted, you may have the opportunity to make the peer review history publicly available. The record will include editor decision letters (with reviews) and your responses to reviewer comments. If eligible, we will contact you to opt in or out.

We look forward to receiving your revised manuscript.

Kind regards,

Yajie Zou
Academic Editor
PLOS ONE

Journal Requirements:

When submitting your revision, we need you to address these additional requirements.

1. Please ensure that your manuscript meets PLOS ONE's style requirements, including those for file naming. The PLOS ONE style templates can be found at

https://journals.plos.org/plosone/s/file?id=wjVg/PLOSOne_formatting_sample_main_body.pdf and

https://journals.plos.org/plosone/s/file?id=ba62/PLOSOne_formatting_sample_title_authors_affiliations.pdf

2. PLOS requires an ORCID iD for the corresponding author in Editorial Manager on papers submitted after December 6th, 2016. Please ensure that you have an ORCID iD and that it is validated in Editorial Manager. To do this, go to ‘Update my Information’ (in the upper left-hand corner of the main menu), and click on the Fetch/Validate link next to the ORCID field. This will take you to the ORCID site and allow you to create a new iD or authenticate a pre-existing iD in Editorial Manager. Please see the following video for instructions on linking an ORCID iD to your Editorial Manager account: https://www.youtube.com/watch?v=_xcclfuvtxQ

[Note: HTML markup is below. Please do not edit.]

Reviewers' comments:

Reviewer's Responses to Questions

**Comments to the Author**

1. Is the manuscript technically sound, and do the data support the conclusions?

The manuscript must describe a technically sound piece of scientific research with data that supports the conclusions. Experiments must have been conducted rigorously, with appropriate controls, replication, and sample sizes. The conclusions must be drawn appropriately based on the data presented.

Reviewer #1: Yes

Reviewer #2: Yes

2. Has the statistical analysis been performed appropriately and rigorously?

Reviewer #1: Yes

Reviewer #2: Yes

3. Have the authors made all data underlying the findings in their manuscript fully available?

The [PLOS Data policy](http://www.plosone.org/static/policies.action#sharing) requires authors to make all data underlying the findings described in their manuscript fully available without restriction, with rare exception (please refer to the Data Availability Statement in the manuscript PDF file). The data should be provided as part of the manuscript or its supporting information, or deposited to a public repository. For example, in addition to summary statistics, the data points behind means, medians and variance measures should be available. If there are restrictions on publicly sharing data—e.g. participant privacy or use of data from a third party—those must be specified.

Reviewer #1: Yes

Reviewer #2: Yes

4. Is the manuscript presented in an intelligible fashion and written in standard English?

PLOS ONE does not copyedit accepted manuscripts, so the language in submitted articles must be clear, correct, and unambiguous. Any typographical or grammatical errors should be corrected at revision, so please note any specific errors here.

Reviewer #1: Yes

Reviewer #2: Yes

5. Review Comments to the Author

Please use the space provided to explain your answers to the questions above. You may also include additional comments for the author, including concerns about dual publication, research ethics, or publication ethics. (Please upload your review as an attachment if it exceeds 20,000 characters)

Reviewer #1: In general, the methods developed in this paper were sound and clear. They investigated multiple parametric methods for firm size and firm age modeling based on grouped data, and proposed a method to estimate the joint distribution of firm size and firm age. The applications of the methods seem interesting.

However, I felt the paper structure was somehow dis-organized.

I suggested the authors have five sections clearly:
(1) Introduction
(2) Materials/Datasets: describe the Legacy BDS Firm Size and Age Data
(3) Methods:
(3.1) Marginal Distribution Estimation Based on Grouped Data
(3.2) Estimating The Joint Distribution of Firm Size and Firm Age
(4) Results
(5) Conclusion

The detailed analysis results in "Estimating The Marginal Distribution of Firm Age" and "Estimating The Marginal Distribution of Firm Size" should be put in the "Results" section.

**We thank the reviewer for the valuable suggestion on restructuring the manuscript. In the revised version, we created a new section named “Materials and dataset” to describe the legacy DBS firm size and age data. The statistical methodology has been kept in a new section named “Methods”.**

**To make the structure more organized, we removed the section title “Marginal Distribution Estimation Based on Grouped Data” and reduced the depth of the subsections of “Fitting A Parametric Model to Grouped Data” and “Goodness-of-fit Assessment” in “Methods” section. The two sub-sections "Estimating The Marginal Distribution of Firm Age" and "Estimating The Marginal Distribution of Firm Size" have been merged with the corresponding subsections in “Results” section.**

Reviewer #2: This paper jointly modeled the firm size and firm age. Some conclusions and phenomena were found. This paper’s topic is interesting, but there are some aspects need to be improved from its current form:
1. The relevant studies in the literature review are not enough for current studies about the topic, some machine learning approaches may be also appropriate for this dataset. For example, see: Evaluation of Short-Term Freeway Speed Prediction Based on Periodic Analysis Using Statistical Models and Machine Learning Models. Journal of Advanced Transportation, 2020.

**Thanks for the valuable comments. We reviewed the paper by Yang et al (2020) and some cited articles. Discussion on the use of machine learning methods to evaluate the impact of periodic component of time has been added to Discussion section.**

2. The problem description section should be added, and the main contribution should be further clearly presented.

**Per the reviewer’s recommendation, description of the problem/ statistical challenges have been discussed and added to the “Introduction” section. More comments on the main contribution have been discussed in both the “Abstract” and the “Conclusion” section. Thanks.**

3. There are some typos in the paper, for example, see Equation (15).

**The typo in Equation (15) has been corrected. In addition, the revised manuscript has been carefully reviewed and proofread by a native English-speaker.**

4. In the results and discussions section, I think that the authors should add more discussion to show the practical significance of the proposed research.

**The main contributions including the practical significance have been discussed in the “Conclusion” section.**

5. None of the explanatory variables are considered in this study to develop the bivariate copula regression model. This issue should also be discussed and what are the potential variables to be included for future research.

**In this manuscript, we focus on univariate and bivariate distribution estimations based on grouped top-coded data. It is a great idea to take into consideration of other explanatory variables in statistical inferences. We briefly discuss this as our potential future research topic. Thanks.**

Therefore, I think this paper is interesting and the above aspects should be further improved.

6. PLOS authors have the option to publish the peer review history of their article ([what does this mean?](https://journals.plos.org/plosone/s/editorial-and-peer-review-process#loc-peer-review-history)). If published, this will include your full peer review and any attached files.


If you choose “no”, your identity will remain anonymous but your review may still be made public.


**Do you want your identity to be public for this peer review?** For information about this choice, including consent withdrawal, please see our [Privacy Policy](https://www.plos.org/privacy-policy).

Reviewer #1: No

Reviewer #2: No


[NOTE: If reviewer comments were submitted as an attachment file, they will be attached to this email and accessible via the submission site. Please log into your account, locate the manuscript record, and check for the action link "View Attachments". If this link does not appear, there are no attachment files to be viewed.]


While revising your submission, please upload your figure files to the Preflight Analysis and Conversion Engine (PACE) digital diagnostic tool, <https://pacev2.apexcovantage.com/>. PACE helps ensure that figures meet PLOS requirements. To use PACE, you must first register as a user. Registration is free. Then, login and navigate to the UPLOAD tab, where you will find detailed instructions on how to use the tool. If you encounter any issues or have any questions when using PACE, please email us at [figures@plos.org](mailto:figures@plos.org). Please note that Supporting Information files do not need this step.
